# Supplementary material for: Retention of paternal DNA methylome in the developing zebrafish germline
Source: Nat Commun. 2019 Jul 11;10:3054. doi: 10.1038/s41467-019-10895-6 (PMC6624265; doi:10.1038/s41467-019-10895-6)
Supplement: Supplementary file 1 — Supplementary Information [file 41467_2019_10895_MOESM1_ESM.pdf]

Retention of paternal DNA methylome in the developing zebrafish germline.
Skvortsova *et al.*

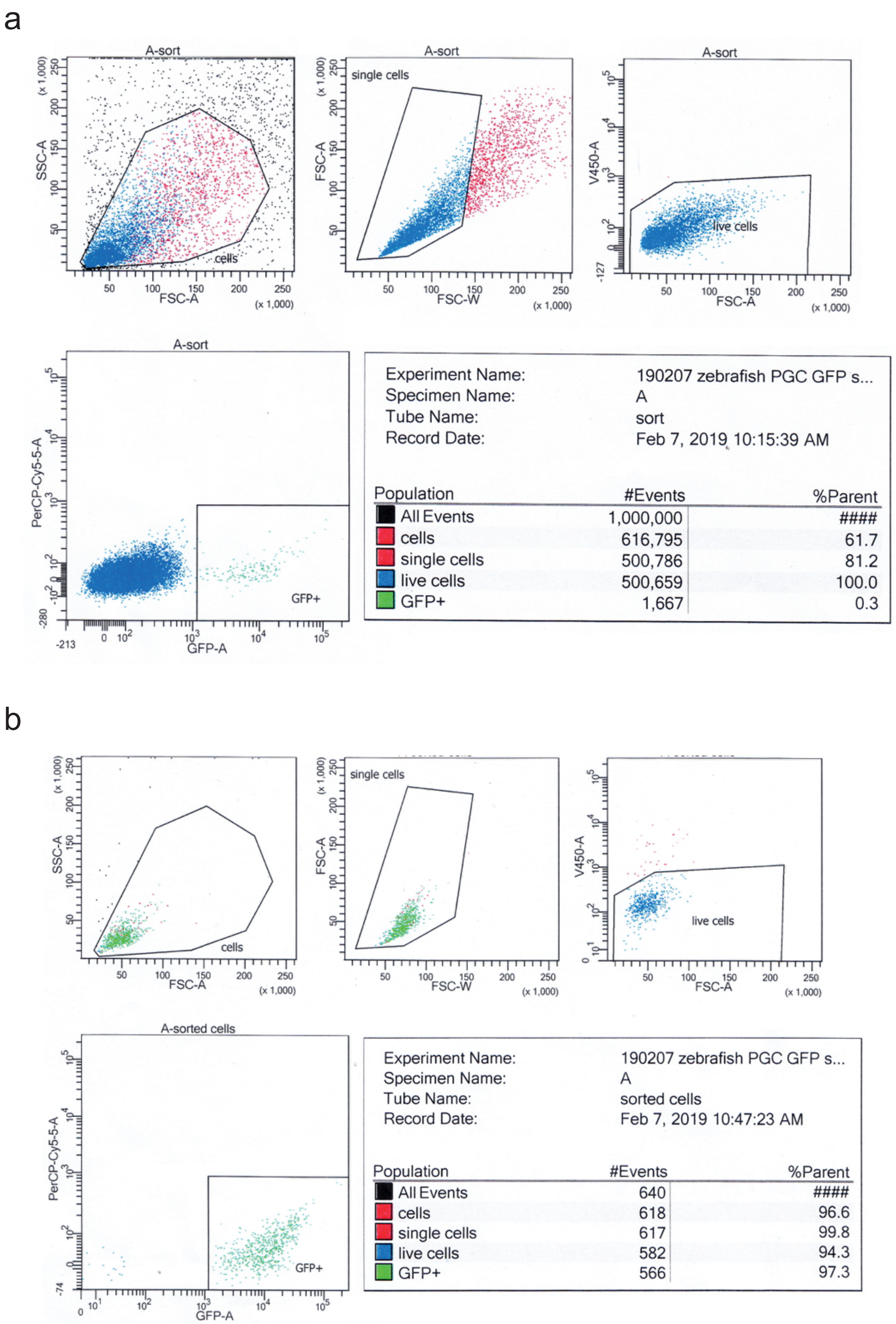

Supplementary Figure 1. FACS gating strategies used for PGC cell sorting. (a) Gating strategy to sort single / live / GFP+ primordial germ cells from the *kop:egfp-f'-nos3'UTR-cry:dsred* zebrafish transgenic line (24hpf). FSC/SSC gating was used to exclude debris and cell clusters and FSC-Area (FSC-A) vs FSC-Width (FSC-W) gating was used to focus on single cells. DAPI was used to exclude dead cells, GFP-positive cells were identified in a SSC vs GFP dot plot. **(b)** Gating strategy to re-sort single / live / GFP+ primordial germ cells from the GFP+ population from panel **(a)** in order to estimate the purity of sorted PGC cells.

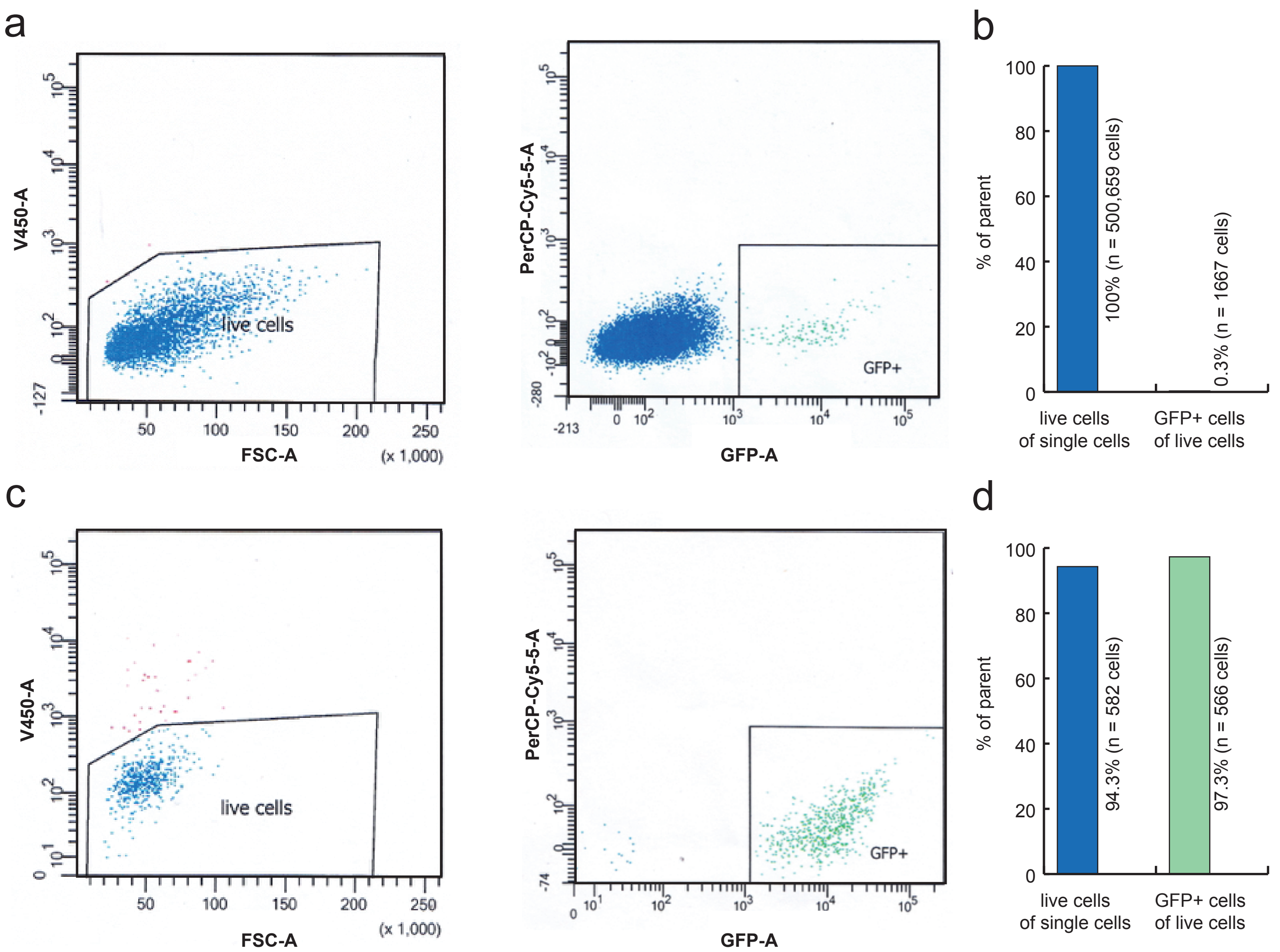

Supplementary Figure 2. Purity estimation of sorted PGC cells (a) FACS gating strategies for the isolation of live (left panel) and live/GFP+ cells (right panel) from the *kop:egfp-f'-nos3'UTR-cry:dsred* zebrafish transgenic line (24hpf). **(b)** Total numbers and percentages of live and live/GFP+ cells after sorting. **(c)** FACS gating strategies for the isolation of live (left panel) and live/GFP+ cells (right panel) after re-sorting of GFP+ from panel **b**. **(d)** Total numbers and percentages of live and live/GFP+ cells after re-sorting of the GFP+ population.

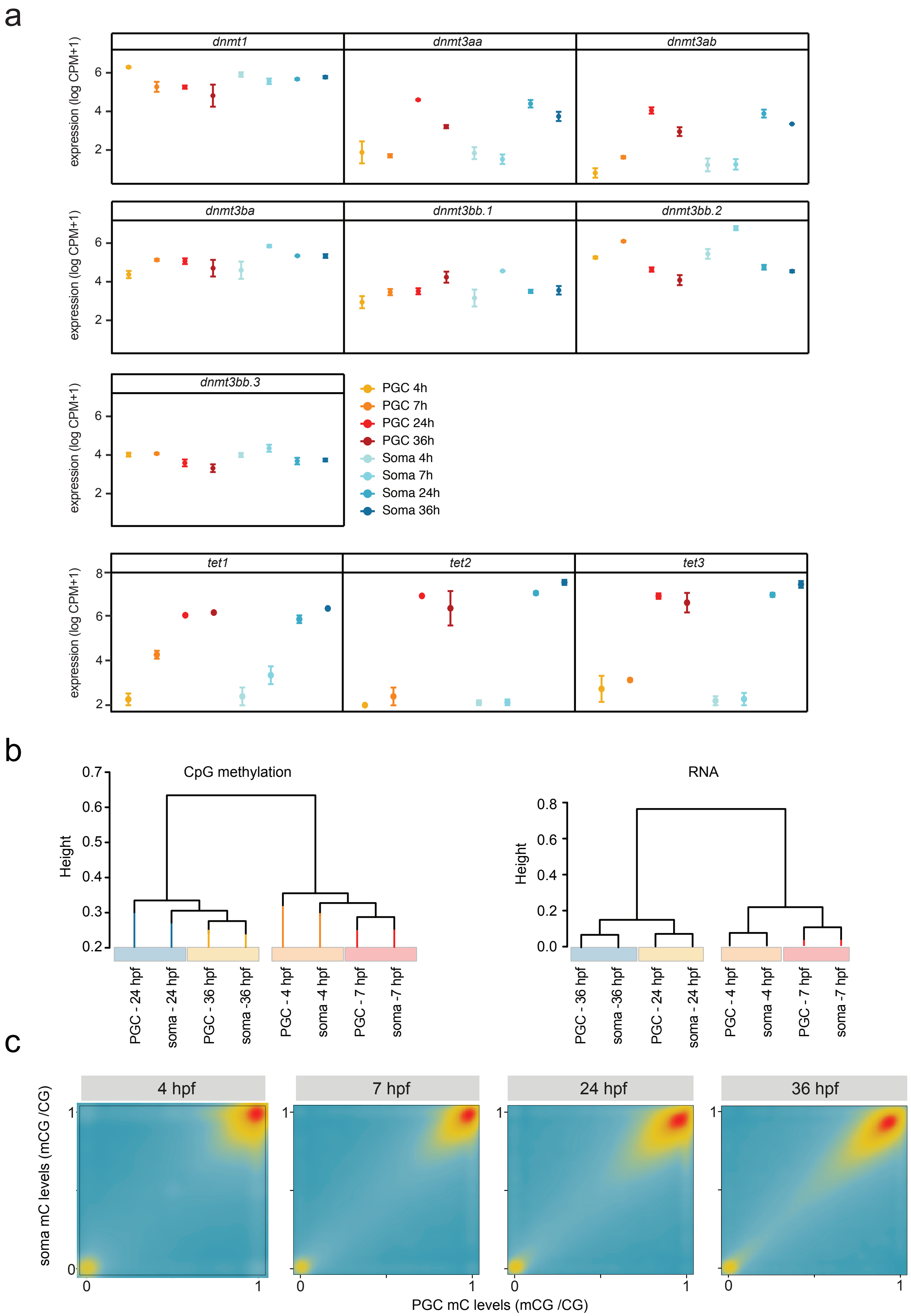

Supplementary Figure 3. 5mC remodelling in zebrafish PGCs and somatic cells (a) Developmental expression patterns of DNA methyltransferases (DNMTs) and TET oxidases in zebrafish PGCs and somatic cells. Expression values are represented as counts per million (log CPM+1). Error bars represent standard error (SE), n = 2 biologically independent replicates. **(b)** Hierarchical clustering of genome-wide DNA methylation (mCG/CG, non-overlapping 1 kb windows) and transcriptome patterns (log CPM+1). **(c)** Global correlation of 5mC levels in non-overlapping 1 kb windows in PGCs and somatic cells. Source data are provided as a Source Data File 6.

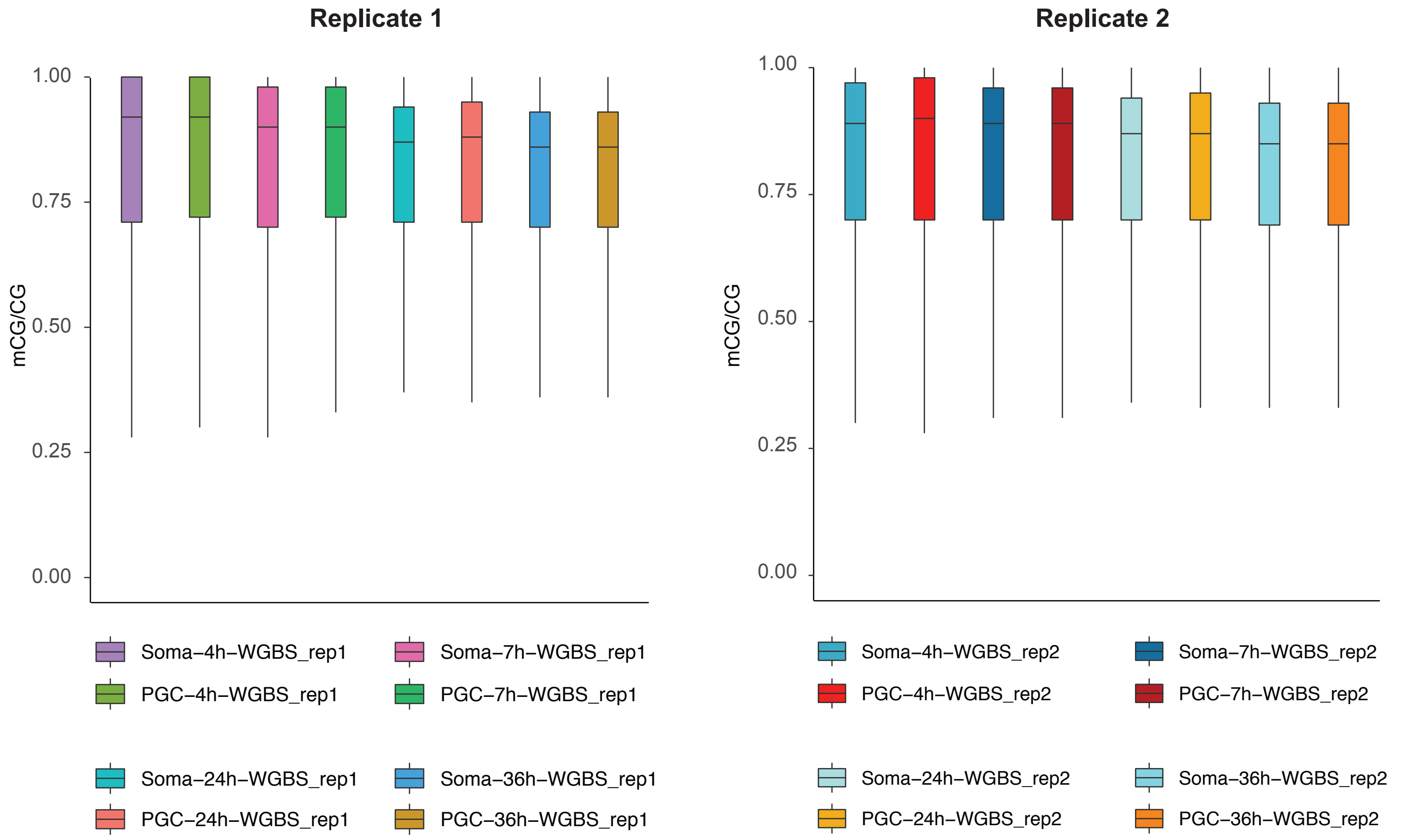

Supplementary Figure 4. Repeat 5mC levels. Boxplots representing 5mC (mCG/CG) values of single CpG sites originating from reads mapping to 2322 consensus repeat sequences from the Repbase database. The boxes show the interquartile range (IQR) around the median. The upper and lower whiskers extend from the hinge to the largest and smallest value, respectively, no further than 1.5 IQR. Outliers are not shown.

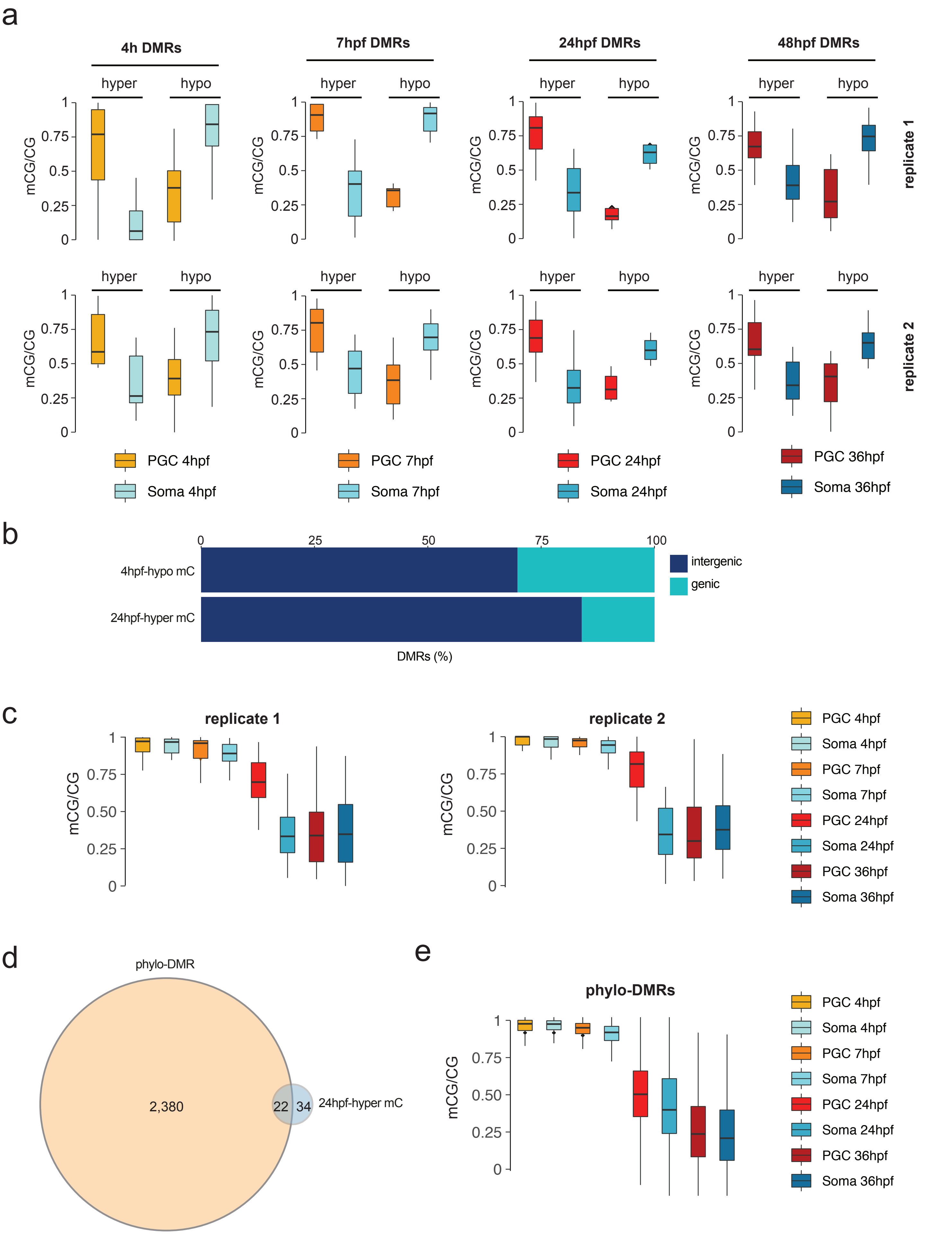

Supplementary Figure 5. Characteristics of DMRs identified between PGCs and somatic cells. (a) Average 5mC levels of 4, 7, 24 and 36 hpf hyper- and hypomethylated DMRs in PGCs and soma. **(b)** Percentage of PGC 4hpf-hypo and 24hpf-hyper DMRs within genic and intergenic regions. **(c)** Dynamics of 5mC at PGC 24hpf-hyper DMRs in PGCs and somatic cells across developmental stages. **(d)** Venn diagram showing an overlap between previously identified phylotypic stage DMRs (phylo-DMRs) and PGC 24hpf-hyper DMRs. **(e)** Dynamics of 5mC at phylo-DMRs in PGCs and somatic cells. The boxes show the interquartile range (IQR) around the median. The upper and lower whiskers extend from the hinge to the largest and smallest value, respectively, no further than 1.5 IQR.

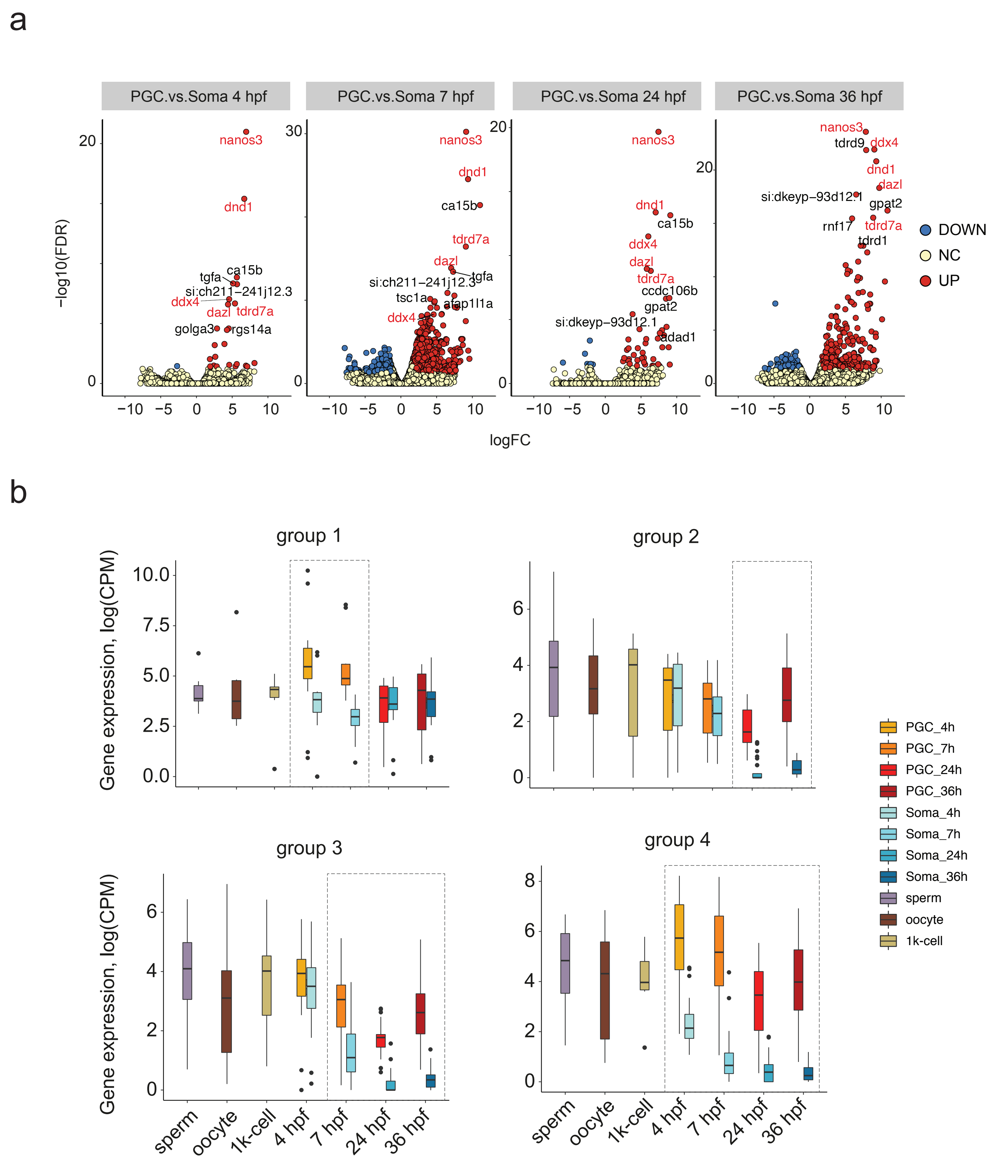

Supplementary Figure 6. Gene expression patterns of PGC-specific genes. (a) Volcano plots showing gene expression differences between PGCs and somatic cells at four developmental stages. Each dot represents a single gene. Genes upregulated in PGCs (logFC > 1.5, FDR < 0.05) are shown as red dots. Genes downregulated in PGCs are shown as blue dots (logFC < -1.5, FDR < 0.05). Genes that are not differentially expressed are shown as yellow dots. Gene symbols of well-established germline markers are indicated in red. Additionally, gene symbols of top five genes ranked by FDR from low to high (except for germline markers) are indicated in black. **(b)** Boxplots showing log transformed CPM expression counts of genes upregulated in PGCs in at least two consecutive developmental stages in zebrafish adult germ cells (sperm, oocyte), 1k-cell embryo and PGCs and somatic cells at four developmental stages. The boxes show the interquartile range (IQR) around the median. The upper and lower whiskers extend from the hinge to the largest and smallest value, respectively, no further than 1.5 IQR.

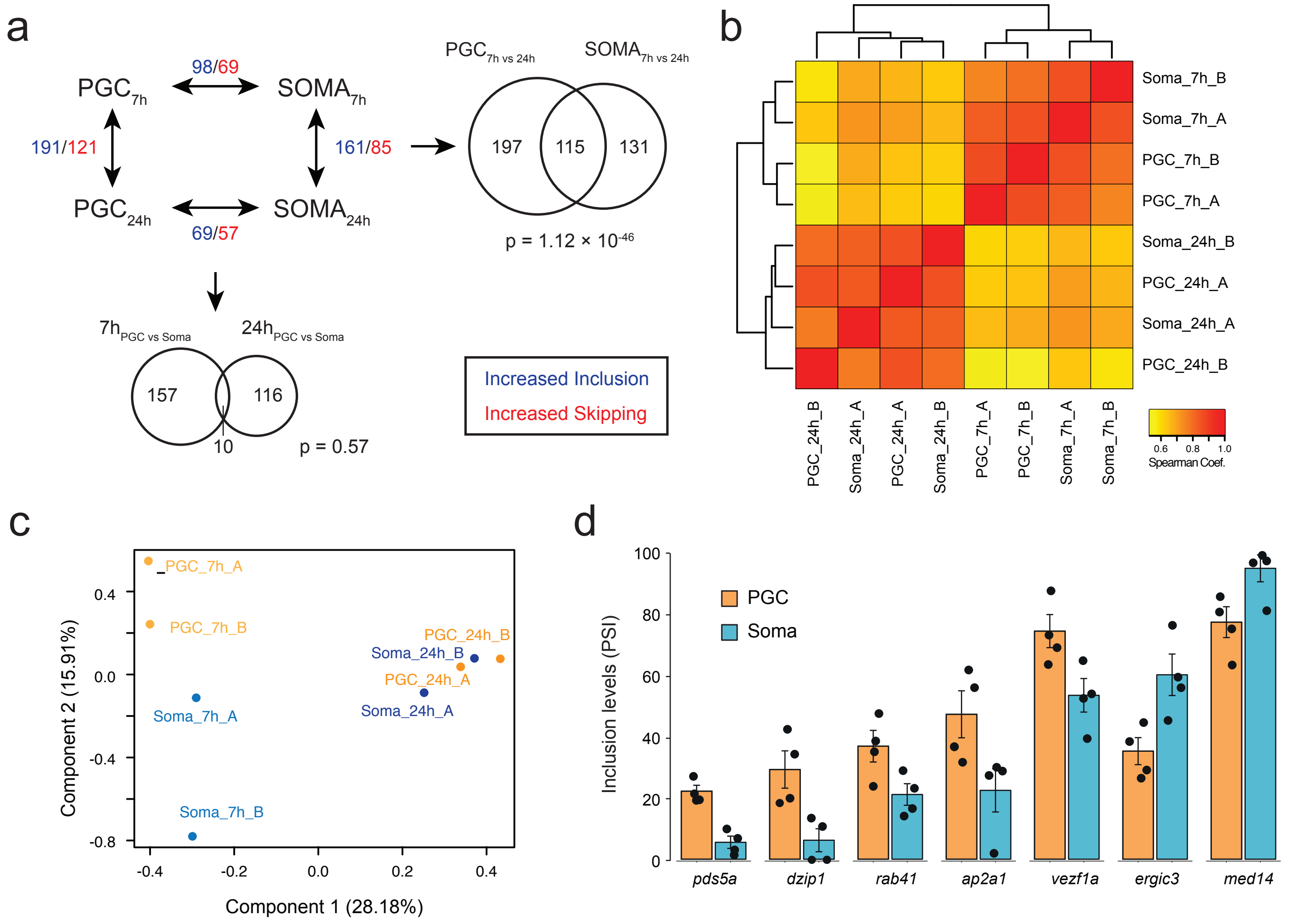

Supplementary Figure 7. Alternative splicing during zebrafish embryogenesis in PGCs and somatic cells (a) Number of exons that show higher (blue) or lower (red) inclusion at any of the studied pairwise comparisons. Overlap between exons that are differentially spliced during development in each tissue (right) and between PGC and soma at each developmental stage (bottom) are shown. p value for the overlaps corresponds to Fisher's exact test. **(b)** Unsupervised clustering of inclusion values for all 498 unique exons that are differentially spliced in any pairwise comparison. Color key corresponds to Spearman correlation coefficient. **(c)** Principal component analysis of exon inclusion levels, showing the first and second component. **(d)** Inclusion levels of the seven most consistently differentially spliced exons between PGCs and soma across developmental stages. Error bars represent standard error (SE), n = 4 biologically independent replicates. Source data are provided as a Source Data File 7.

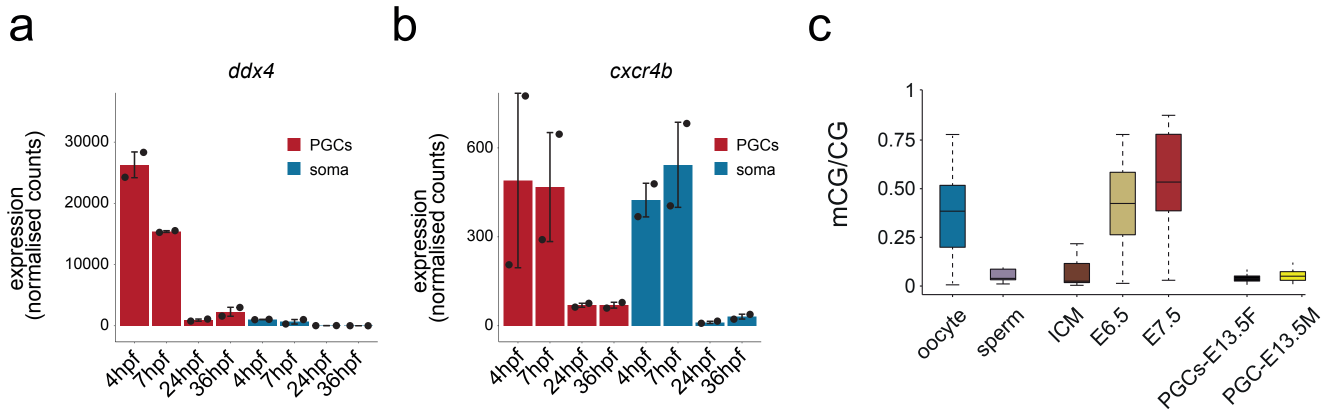

Supplementary Figure 8. 5mC targeting of germline gene promoters in zebrafish and mouse embryos. (a, b) Developmental expression dynamics of *dazl* **(a)** and *cxcr4b* **(b)** in PGCs and somatic cells across four developmental stages. Error bars represent standard error (SE), n = 2 biologically independent replicates. **(c)** Boxplots of 5mC in promoter-associated CpG islands (Bio-CAP) in mouse adult germ cells (oocyte, sperm), early embryos, and developing PGCs. The boxes show the interquartile range (IQR) around the median. The upper and lower whiskers extend from the hinge to the largest and smallest value, respectively, no further than 1.5 IQR. Source data are provided as a Source Data File 8.

a

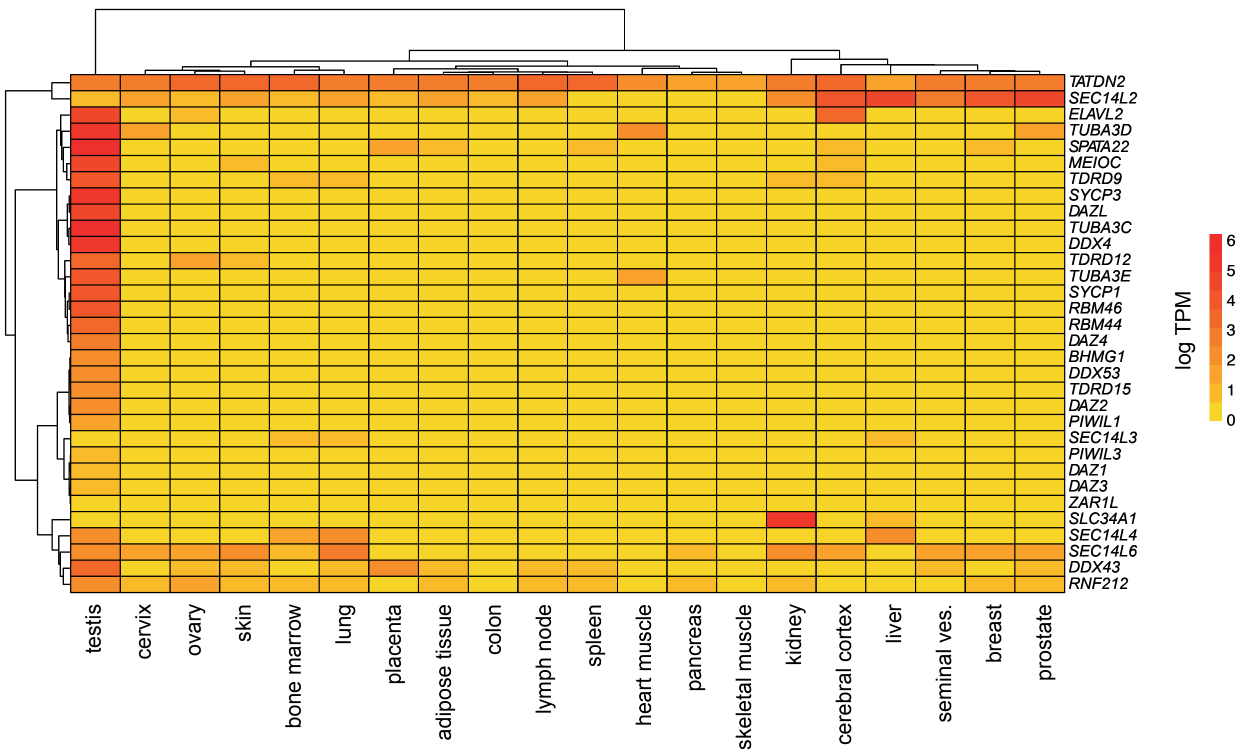

b

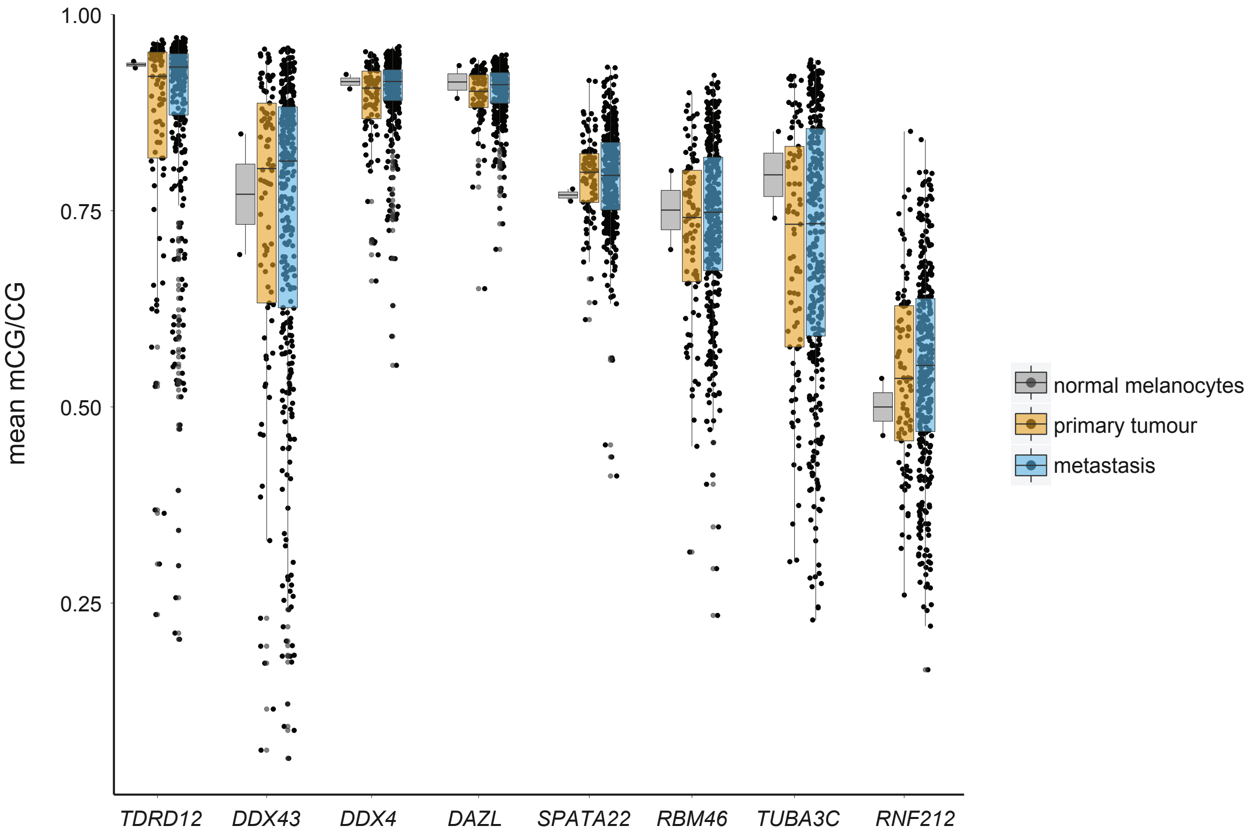

Supplementary Figure 9. Gene expression in different human tissues and DNA methylation status in melanoma of evolutionarily conserved promoter 5mC targets. (a) Heatmap of log-transformed TPM expression values of conserved promoter 5mC targets in human tissues. **(b)** Boxplots showing promoter 5mC in the TCGA Skin Cutaneous Melanoma (TCGA-SKCM) patient samples (primary tumour and metastasis compared to normal melanocytes). Each dot represents a single patient. 5mC data of TCGA-SKCM samples in the form of methylation beta values (derived from the HumanMethylation 450K beadChIP arrays) were downloaded from the National Cancer Institute Genomic Data Commons (GDC) Legacy Archive repository. The boxes show the interquartile range (IQR) around the median. The upper and lower whiskers extend from the hinge to the largest and smallest value, respectively, no further than 1.5 IQR. Source data are provided as a Source Data File 9.
