# Supplementary material for: Retention of paternal DNA methylome in the developing zebrafish germline
Source: Nat Commun. 2019 Jul 11;10:3054. doi: 10.1038/s41467-019-10895-6 (PMC6624265; doi:10.1038/s41467-019-10895-6)
Supplement: Supplementary file 3 — Description of Additional Supplementary Files [file 41467_2019_10895_MOESM3_ESM.pdf]

Description of Additional Supplementary Files

File name: Supplementary Data 1

Description: WGBS metrics

File name: Supplementary Data 2

Description: genomic position of PGC-soma DMRs overlapping the Repeatmasker track

File name: Supplementary Data 3

Description: genomic position of PGC-soma DMRs not overlapping the Repeatmasker track

File name: Supplementary Data 4

Description: GO enrichment (GREAT) for 24hpf hypermC DMRs

File name: Supplementary Data 5

Description: RNA-seq mapping metrics

File name: Supplementary Data 6

Description: GO terms associated with genes upregulated in 4h PGC sample

File name: Supplementary Data 7

Description: GO terms associated with genes upregulated in 7h PGC sample

File name: Supplementary Data 8

Description: GO terms associated with genes upregulated in 24h PGC sample

File name: Supplementary Data 9

Description: GO terms associated with genes upregulated in 36h PGC sample

File name: Supplementary Data 10

Description: GO terms associated with genes upregulated in all PGC samples

File name: Supplementary Data 11

Description: GO terms associated with genes upregulated in PGC samples in zebrafish, mouse, and human

File name: Supplementary Data 12

Description: GO enrichments of genes alternatively spliced between PGCs and soma at 7 hpf

File name: Supplementary Data 13

Description: GO enrichments of genes alternatively spliced between PGCs and soma at 24 hpf

File name: Supplementary Data 14

Description: 5mC levels in developmentally methylated soma/PGC promoters

File name: Supplementary Data 15

Description: GO enrichment of developmental 5mC target genes
