# Supplementary material for: Retention of paternal DNA methylome in the developing zebrafish germline
Source: Nat Commun. 2019 Jul 11;10:3054. doi: 10.1038/s41467-019-10895-6 (PMC6624265; doi:10.1038/s41467-019-10895-6)
Supplement: Supplementary file 19 — Reporting Summary [file 41467_2019_10895_MOESM19_ESM.pdf]

nature research

Corresponding author(s): Ozren Bogdanovic

Last updated by author(s): May 17, 2019

Reporting Summary

Nature Research wishes to improve the reproducibility of the work that we publish. This form provides structure for consistency and transparency in reporting. For further information on Nature Research policies, see Authors & Referees and the Editorial Policy Checklist.

Statistics

For all statistical analyses, confirm that the following items are present in the figure legend, table legend, main text, or Methods section.

n/a	Confirmed	
☐	☒	The exact sample size (*n*) for each experimental group/condition, given as a discrete number and unit of measurement
☐	☒	A statement on whether measurements were taken from distinct samples or whether the same sample was measured repeatedly
☐	☒	The statistical test(s) used AND whether they are one- or two-sided<br>*Only common tests should be described solely by name; describe more complex techniques in the Methods section.*
☐	☒	A description of all covariates tested
☐	☒	A description of any assumptions or corrections, such as tests of normality and adjustment for multiple comparisons
☐	☒	A full description of the statistical parameters including central tendency (e.g. means) or other basic estimates (e.g. regression coefficient) AND variation (e.g. standard deviation) or associated estimates of uncertainty (e.g. confidence intervals)
☐	☒	For null hypothesis testing, the test statistic (e.g. *F*, *t*, *r*) with confidence intervals, effect sizes, degrees of freedom and *P* value noted<br>*Give P values as exact values whenever suitable.*
☒	☐	For Bayesian analysis, information on the choice of priors and Markov chain Monte Carlo settings
☒	☐	For hierarchical and complex designs, identification of the appropriate level for tests and full reporting of outcomes
☐	☒	Estimates of effect sizes (e.g. Cohen's *d*, Pearson's *r*), indicating how they were calculated

Our web collection on statistics for biologists contains articles on many of the points above.

Software and code

Policy information about availability of computer code

Data collection

N/A

Data analysis

WGBS MAPPING
• read trimming: fastp v0.12.5 (https://github.com/OpenGene/fastp); Trimmomatic v0.38 (http://www.usadellab.org/cms/?page=trimmomatic)
• mapping: WALT v1.0 (https://github.com/smithlabcode/walt)
• SAM/BAM manipulation: SAMtools v1.6 (https://github.com/samtools/samtools); Picard-tools v2.3.0 ("Picard Toolkit." 2019. Broad Institute, GitHub Repository. http://broadinstitute.github.io/picard/; Broad Institute)
• methylation calling: MethylDackel v0.3.0 (https://github.com/dpryan79/MethylDackel)

Zebrafish PGC and soma DNA methylome libraries were sequenced on the Illumina HiSeq X platform (high throughput mode, 150 bp, PE), generating an average of 75M reads per sample. Sequenced reads in FASTQ format were trimmed using the fastp tool v0.12.5 with the following settings: (fastp -i ${read_1} -I ${read_2} -o ${trimmed_read_1} -O ${trimmed_read_2} -f 10 -t 10 -F 10 -T 10), This step removes potential adapter sequences and trims 10 bp of the 5' and 3' end of each read (required due to the addition of adaptase tails during library preparation). Trimmed reads were mapped (danRer10 genome reference, containing the lambda genome as chrLambda) using WALT with the following settings: -m 10 -t 24 -N 10000000 -L 2000. Mapped reads in SAM format were converted to BAM format; BAM files were merged, sorted and indexed using SAMtools. PCR and optical duplicates were removed using Picard tools v2.3.0. Genotype and methylation bias correction was performed using MethylDackel (see parameters –minOppositeDepth, --maxVariantFrac and –OT, --OB below). The number of methylated and unmethylated calls at each genomic CpG position were determined using MethylDackel (MethylDackel extract genome_lambda.fa $input_bam -o output --mergeContext --minOppositeDepth 5 --maxVariantFrac 0.5 --OT 0,120,0,120 --OB 10,0,10,0). Adult liver WGBS methylome was mapped with the same settings, however, trimming was performed using the Trimmomatic software without the hard-trimming step (ILLUMINACLIP:adapter.fa:2:30:10 SLIDINGWINDOW:5:20 LEADING:3 TRAILING:3 MINLEN:50), as standard library prep doe not require this type of filtering. To assess the mC abundance and dynamics at repetitive elements, sequencing reads were using the fastp v0.12.5 and mapped using WALT v1.0, as described above, to danRer10 repeat-masked genome combined with an in silico reference of canonical repeat sequences, each being represented as a single copy. The

number of methylated and unmethylated calls at each genomic CpG position were called using the MethylDackel v0.3.0 with the following settings: (MethylDackel extract --keepSingleton --keepDiscordant genome_lambda.fa $input_bam -o output --mergeContext). Differentially methylated CpG sites between PGCs and soma at each developmental stage were identified using DMLtest function of the DSS package (https://github.com/haowulab/DSS/blob/master/R/DML.R). For 7, 24 and 36 hpf time points DMLtest was performed using biological replicates per each time point. For 4 hpf time point DMLtest was performed separately for each technical replicate with the commonly identified differentially methylated CpGs being used in the downstream analyses. Differentially methylated CpG sites located within 50 bp from each other were joined into regions (DMRs), which were filtered to harbour at least 5 differentially methylated CpGs and span at least 50 bp. DMRs overlapping repetitive regions (RepeatMasker) and/or containing more than 25% of CpGs within them with sequencing coverage less than 5x were excluded from the analysis. The remaining DMRs were defined as hypomethylated or hypermethylated when both replicates displayed at least 10% average methylation difference between PGCs and soma.
RNA-seq MAPPING
• read trimming: TrimGalore v0.4.0 (https://github.com/FelixKrueger/TrimGalore)
• mapping: using STAR v2.4.0d (https://github.com/alexdobin/STAR)
• SAM/BAM manipulation: SAMtools v1.1 (https://github.com/samtools/samtools)
• read counting: RSEM v1.2.21 (https://github.com/deweylab/RSEM)
• Differential gene expression analyses: edgeR v3.24.0 (https://bioconductor.org/packages/release/bioc/html/edgeR.html)

Zebrafish PGC and soma rRNA-depleted RNA libraries were sequenced on the Illumina NovaSeq 6000 platform (high throughput mode, 100 bp, PE). Illumina Adapters and Pico v2 SMART adapter trimming (first three nucleotides of R2) was performed using TrimGalore with the following settings: (trim_galore --paired --clip_R2 3). Trimmed sequencing reads were aligned to the reference zebrafish genome danRer10 using STAR with the following settings: (STAR --runMode alignReads --runThreadN 6 --genomeLoad LoadAndKeep --readFilesCommand zcat --outFilterType BySJout --outSAMattributes NH HI AS NM MD --outFilterMultimapNmax 20 --outFilterMismatchNmax 999 --outFilterMismatchNoverReadLmax 0.04 --alignIntronMax 1500000 --alignMatesGapMax 1500000 --alignIntronMin 20 --alignSJoverhangMin 6 --alignSJDBoverhangMin 1 --outFilterMatchNmin 99 --quantMode TranscriptomeSAM --outSAMtype BAM Unsorted). Mapped reads in SAM format were sorted and indexed using SAMtools. Quantification of transcript abundances was performed using RSEM with the following settings: (rsem-calculate-expression --paired-end --bam --no-bam-output --seed 12345 -p 6 --forward-prob 0). Differential gene expression analysis was performed using edgeR v3.24.0. GENE_PGCs_Soma_expected_count_table.csv provided as GEO series GSE122480 was used to calculate gene expression log(CPM+1) values. First, rows with ≤ 3 raw read counts were filtered out. Filtered table of counts was converted into a DGEList object using DGEList() {edgeR} function. The calcNormFactors() {edgeR} function was used to normalize for RNA composition using the trimmed mean of M-values (TMM) method to compute the scaling factors. Counts per million for each gene were calculated using cpm() {edgeR} function. A negative binomial generalized log-linear model (GLM) was fitted to the read counts for each gene using glmFit() {edgeR} function. Likelihood ratio tests (LRT) for PGC vs soma for each developmental stage were conducted using glmLRT() {edgeR} function. Benjamini-Hochberg method was used to adjust p-values for multiple testing. Differentially expressed genes were extracted using topTags() {edgeR} function. Genes that displayed ±1.5 logFC (FDR < 0.05) between PGCs and soma samples were considered as significantly differentially expressed and were used in the downstream analysis. The downstream data analysis was performed using R (R version 3.5.0 (2018-04-23); Platform: x86_64-apple-darwin15.6.0 (64-bit); Running under: macOS Sierra 10.12.6) and deepTools2 functions specified below.

DOWNSTREAM ANALYSIS

Figure 1a. ImageJ (https://imagej.nih.gov/ij/download.html).
Figure 2a. geom_point() and geom_errorbar() functions {ggplot2 v3.1.0} was used to plot log(CPM+1) gene expression values (see above) of key germline genes at 4 hpf. Errorbars represent standard error (SE). Log2-fold change of gene expression between PGC and soma is plotted using geom_bar() function {ggplot2 v3.1.0}.
Figure 2b. Histograms of single CpG 5mC levels were plotted using geom_histogram(binwidth=0.1) function {ggplot2 v3.1.0}. Only CpGs with sequencing coverage ≥ 5 in all the samples were used. CpG methylation tables (replicate 1 and 2) are provided as GEO series GSE122722.
Figure 2c. Principal component analysis of embryonic PGCs, corresponding somatic cells, and whole embryo methylomes (256-cell embryos SRP020008) as well as adult germline (oocyte (1), sperm (1) GSE44075; oocyte (2), sperm (2) SRP020008) and somatic methylomes (adult brain GSE68087; adult liver-this study) was performed using prcomp() R function and plotted using geom_point() function {ggplot2 v3.1.0}. Average 5mC values were calculated for 10 kbp non-overlapping bins across the genome for each sample using overlapRatios() function (https://github.com/astatham/aaRon/blob/master/R/overlaps.R).
Figure 2d. Boxplots were generated using geom_boxplot() function {ggplot2 v3.1.0}. The zebrafish danRer10 genome was binned into equally-sized consecutive 10 kb-wide bins using tileGenome() function {GenomicRanges v1.34.0}. Average methylation was calculated for each bin and plotted using geom_boxplot() function for each sample. The samples shown are described in Figure 2c.

Figure 3a. Alluvial plots were generated using geom_flow() function {ggalluvial v0.9.1}. PGC-soma hypo- and hypermethylated DMRs at four developmental stages are provided as a Supplementary Table 2.
Figure 3b. PGC/soma WGBS (this study) and BioCAP (GSE43512) data was visualized using Integrative Genomics Viewer (IGV) v2.4.4.
Figure 3c, d. Boxplots were generated using geom_boxplot() function {ggplot2 v3.1.0}. H3K4me1 and H3K27ac ChIP-seq data from zebrafish dome and 24 hpf stage embryos (GSE32483) and BioCAP data from zebrafish testis, liver and 24 hpf embryos (GSE43512) was mapped to danRer10 zebrafish reference genome using bowtie v1.1.0. BioCAP signal enrichment relative to the input in the form of log2ratio was calculated using bamCompare function (RPKM normalization mode) {deepTools2}. log2ratio scores per DMR were calculated using overlapMeans() function (https://github.com/astatham/aaRon/blob/master/R/overlaps.R). H3K4me1/H3K27ac enrichment over DMRs was calculated using bamCoverage function (RPKM normalization mode) {deepTools2}. RPKM values per DMR were calculated using overlapMeans() function (https://github.com/astatham/aaRon/blob/master/R/overlaps.R). Control regions were selected randomly from the zebrafish danRer10 genome using bedtools shuffle function {bedtools v2.22.0}. Statistical hypothesis testing was performed using the t.test function ("two.sided", "less", "greater", mu = 0, paired = FALSE, var.equal = FALSE, conf.level = 0.95)
Figure 3e. Gene ontology analysis was performed using GREAT v3.0.0.
Figure 3f. 5hmC signal (GSE68087) over DMRs (extended 3 kbp from the centre) was calculated using computeMatrix reference-point (bin size 300 bp) and plotted using plotProfile() function {deepTools2}. Wilcoxon signed rank test was performed usin the "wilcox.test" function with default settings t(x, y = NULL, alternative = c("two.sided", "less", "greater"), mu = 0, paired = FALSE, exact = NULL, correct =

TRUE, conf.int = FALSE, conf.level = 0.95)

Figure 4a. Gene ontology analysis was performed using g:Profiler tool (https://biit.cs.ut.ee/gprofiler/index.cgi)
Figure 4b. Heatmap was generated using pheatmap() function {pheatmap v1.0.10}. Pearson correlation was used as a distance measure, ward.D2 was used as clustering method. Only genes upregulated (logFC > 1.5, FDR < 0.05) in PGC compared to soma in at least two consecutive developmental stages were used in the analysis.
Figure 4c-f. For genes upregulated at minimum two consecutive developmental stages in zebrafish PGCs (see Figure 4b), mouse and human homologues were identified using the ENSEMBL BioMART tool. Mouse single cell RNA-seq data for E11.5, E13.5 female, E16.5 female and E16.5 male PGCs and corresponding soma samples was downloaded from GSE79552. Human single cell RNA-seq data for 4W, 7W, 8W, 11W male/female and 17W female and 19W male embryos containing the highest number of sequenced single PGCs and corresponding somatic cells was downloaded from GSE63818. Mean FPKM values across single cells for each sample were calculated and plotted in a log transformed format per each gene. Boxplots (c, d) were generated using geom_boxplot() function {ggplot2 v3.1.0}. Statistical hypothesis testing was performed using the t.test function ("two.sided", "less", "greater", mu = 0, paired = FALSE, var.equal = FALSE, conf.level = 0.95) Heatmaps (e, f) were generated using pheatmap() function{pheatmap v1.0.10}.

Figure 5a. DNA methylation heatmaps of novel putative 5mC-regulated NMI promoters were generated using pheatmap() function {pheatmap v1.0.10}. NMI peaks were called by MACS2 (narrowPeak) separately for zebrafish testis, liver and 24 hpf embryo samples. Then, the NMI peaks was merged and overlapped with GRCz10/danRer10 Ensembl gene transcription start sites. Average CpG methylation was calculated for each NMI in soma samples across four developmental stages. NMIs with Pearson correlation coefficients between 5mC and developmental stages > 0.5 (indicative of a gradual increase of 5mC in somatic cells across stages) with 4 hpf 5mC < 0.025 and 36 hpf 5mC > 0.15 in each replicate and displaying > 0.45 5mC in adult zebrafish brain and liver tissues were defined as putative 5mC targets and their methylation values were plotted. Additionally, egg (GSE44075), sperm (GSE44075), adult brain (GSE68087) and adult liver (this study) DNA methylation was calculated and plotted for the selected set of NMIs.
Figure 5b. Boxplots were generated R boxplot function {ggplot2 v3.1.0}. Raw gene expression counts (RSEM) were first multiplied by scaling factors to account for read mapping depth differences. Such caled reads were then normalised using RUVseq package (median normalisation).
Figure 5c. PGC/soma WGBS (this study) and oocyte/sperm WGBS (GSE44075) data was visualized using Integrative Genomics Viewer (IGV) v2.4.4.
Figure 5d. DNA methylation heatmaps of novel putative 5mC-regulated NMI promoters were generated using pheatmap() function {pheatmap v1.0.10}. Zebrafish gene IDs were first converted into mouse homologues using an ENSEMBL BioMart tool. Transcription start sites of homologous mouse genes were overlapped with mouse NMIs (merged testis, liver and ESC BioCAP peaks) and average 5mC was calculated per NMI. Mouse sperm, oocyte, early embryo (ICM, E6.5, E7.5) and PGCs (E13.5 female and E13.5 male) (GSE56697) WGBS DNA methylation data was used.
Figure 5e. Scatterplots of promoter NMIs DNA methylation vs corresponding gene expression data were generated using geom_point() function {ggplot2 v3.1.0}. Linear regression line was plotted using geom_smooth(method = "lm", se = TRUE) function {ggplot2 v3.1.0}. CpG methylation data of TCGA Skin Cutaneous Melanoma samples (TCGA-SKCM) in the form of methylation beta values (derived from the HumanMethylation 450K beadChIP arrays) was downloaded from the National Cancer Institute Genomic Data Commons (GDC) Legacy Archive repository. Gene expression data of TCGA-SKCM samples in the form of mRNA Expression z-Scores (RNA-seq V2 RSEM) was downloaded from cBioPortal. For each promoter NMI (merged BioCAP peaks from human testis and liver that overlapped hg19 Ensembl gene transcription start sites) the methylation score was calculated as the mean methylation beta value of all CpG probes overlapping the NMI. These methylation scores were plotted against the mRNA expression z-scores of corresponding genes.

For manuscripts utilizing custom algorithms or software that are central to the research but not yet described in published literature, software must be made available to editors/reviewers. We strongly encourage code deposition in a community repository (e.g. GitHub). See the Nature Research guidelines for submitting code & software for further information.

Data

Policy information about availability of data

All manuscripts must include a data availability statement. This statement should provide the following information, where applicable:

- Accession codes, unique identifiers, or web links for publicly available datasets
- A list of figures that have associated raw data
- A description of any restrictions on data availability

All DNA methylation (WGBS) and expression (RNA-seq) data have been made available through the Gene Expression Omnibus (GEO). The following secure token has been created to allow review of record GSE122723 while it remains in private status: efofmkscxtslryp. The data will be made publicly available upon publication of the manuscript.

Field-specific reporting

Please select the one below that is the best fit for your research. If you are not sure, read the appropriate sections before making your selection.

☒ Life sciences ☐ Behavioural & social sciences ☐ Ecological, evolutionary & environmental sciences

For a reference copy of the document with all sections, see nature.com/documents/nr-reporting-summary-flat.pdf

Life sciences study design

All studies must disclose on these points even when the disclosure is negative.

Sample size	Each RNA-seq / WGBS sample was prepared from 10,000 - 14,000 cells.
Data exclusions	N/A
Replication	Each WGBS and RNA-seq sample was prepared in biological replicates (2 X), except for WGBS (soma and PGC) sample extracted at 4h which is a technical replicate (2X - two independent library preparations from the same DNA).
Randomization	N/A
Blinding	N/A

Reporting for specific materials, systems and methods

We require information from authors about some types of materials, experimental systems and methods used in many studies. Here, indicate whether each material, system or method listed is relevant to your study. If you are not sure if a list item applies to your research, read the appropriate section before selecting a response.

Materials & experimental systems

n/a	Involved in the study	
☒	☐	Antibodies
☒	☐	Eukaryotic cell lines
☒	☐	Palaeontology
☐	☒	Animals and other organisms
☒	☐	Human research participants
☒	☐	Clinical data

Methods

n/a	Involved in the study	
☒	☐	ChIP-seq
☐	☒	Flow cytometry
☒	☐	MRI-based neuroimaging

Animals and other organisms

Policy information about studies involving animals; ARRIVE guidelines recommended for reporting animal research

Laboratory animals	Zebrafish (Danio rerio) transgenic line Tg(kop:egfp-f'-nos3'UTR-cry:dsred) was used for the preparation of PGC/soma (4h, 7h, 24h, 36h) WGBS and RNA-seq samples. Zebrafish liver WGBS sample was obtained from an adult female (AB strain).
Wild animals	N/A
Field-collected samples	N/A
Ethics oversight	The general fish maintenance at the Institute follows the regulations of the LANUV NRW and is supervised by the veterinarian office of the city of Muenster (Germany).

Note that full information on the approval of the study protocol must also be provided in the manuscript.

Flow Cytometry

Plots

Confirm that:

☒ The axis labels state the marker and fluorochrome used (e.g. CD4-FITC).

☒ The axis scales are clearly visible. Include numbers along axes only for bottom left plot of group (a 'group' is an analysis of identical markers).

☒ All plots are contour plots with outliers or pseudocolor plots.

☒ A numerical value for number of cells or percentage (with statistics) is provided.

Methodology

Sample preparation	In brief, cells were dissociated with enzyme free dissociation buffer (BD), washed with Ca/Mg free PBS, and then filtered through a 40-μm nylon mesh to remove large cell clusters. GFP-positive cells were isolated by Fluorescent Activated Cell Sorting (FACS) on a FACSAria IIIu cell sorter with FACSDiva software (BD Biosciences) using a 70 μm nozzle.

Instrument	FACSAria III cell sorter (BD Biosciences) equipped with a 70-µm nozzle.
Software	FACSDiva software (BD Biosciences) operating the cell sorter. Data analysis was done using FlowJo software.
Cell population abundance	Cell population abundance: see tables - 0.3% of PGCs as an initial GFP + target cell fraction. Purity of the samples was determined by re-sorting of the sorted GFP+ (PGC) population. The purity in the resorting experiment was 97.3%
Gating strategy	We used FSC/SSC gating to exclude debris and cell clusters and FSC-Area vs FSC-Width gating to focus on single cells. DAPI was used to exclude dead cells, GFP-positive cells were identified in a SSC vs GFP dot plot.

☒ Tick this box to confirm that a figure exemplifying the gating strategy is provided in the Supplementary Information.
